# The Efficacy of Cardiac Anti-miR-208a Therapy Is Stress Dependent

**DOI:** 10.1016/j.ymthe.2017.01.012

**Published:** 2017-02-12

**Authors:** Joep E.C. Eding, Charlotte J. Demkes, Joshua M. Lynch, Anita G. Seto, Rusty L. Montgomery, Hillary M. Semus, Aimee L. Jackson, Marc Isabelle, Stefano Chimenti, Eva van Rooij

**Affiliations:** 1 Hubrecht Institute, KNAW and University Medical Center Utrecht, 3584CT Utrecht, the Netherlands; 2 Department of Cardiology, University Medical Center Utrecht, 3584CT Utrecht, the Netherlands; 3 miRagen Therapeutics, Inc., Boulder, CO 80301, USA; 4 Servier Research Institute, Suresnes 92150, France

**Keywords:** microRNA, anti-miR, target

## Abstract

MicroRNAs (miRNAs) are important regulators of biology and disease. Recent animal efficacy studies validate the therapeutic benefit of miRNA modulation and underscore the therapeutic value of miRNA-targeting oligonucleotides. However, whether disease conditions (stress) influence the pharmacological effects of an anti-miR is currently unknown. To study the effect of disease on target regulation after anti-miR treatment, we injected animals with anti-miR-208a, a synthetic oligonucleotide that inhibits the cardiomyocyte-specific miR-208a. Our data indicate that the presence of stress increases the number of regulated miR-208a targets, and that higher stress levels correlate with stronger target derepression. Additionally, the type of stress also influences which targets are regulated upon miR-208a inhibition. Studies in a large animal model indicate a similar stress-dependent anti-miR effect. Subsequent in vitro studies suggest that the influence of stress on anti-miR efficacy depends at least in part on increased cellular anti-miR uptake. These data indicate that the pharmacological effect of anti-miRs is stronger under disease conditions, and that both the type and severity of disease determine the therapeutic outcome. These facts will be important for assessing the therapeutic dose and predicting the therapeutic outcome when applying anti-miRs in a clinical setting.

## Teaser

MicroRNA inhibitors, known as anti-miRs, can function to target disease-related microRNAs. In this issue of *Molecular Therapy*, Eding et al. show that the efficacy of anti-miR-208a is dependent on the presence of stress. This is partially explained by an increase in cellular uptake of the anti-miR under disease conditions.

## Introduction

MicroRNAs (miRNAs) are short, single-stranded RNAs that anneal with complementary sequences in target mRNAs, thereby suppressing protein formation. The function of a given miRNA is determined by its mRNA targets.^1^ Because an individual miRNA can engage numerous mRNA targets, often encoding multiple components of complex intracellular networks, the regulation of a single miRNA can have a profound impact on cellular phenotypes.^2^ It is well accepted that miRNAs are important regulators of biology and disease. Their obvious relevance in disease, as well as their known conserved sequence, catalyzed efforts to explore miRNAs as novel drug targets. Antisense chemistries, known as anti-miRs, can function to target disease-related miRNAs in vivo. They can reduce the levels of pathogenic or aberrantly expressed miRNAs^1^, ^3^, ^4^ and are efficacious in both animals and humans.^2^, ^5^ Because miRNAs typically act as inhibitors of gene expression, anti-miRs will derepress translation of the mRNAs that are normally targeted by the miRNA.^3^ Previously, we reported that systemic delivery of an antisense oligonucleotide against miR-208a induced potent and sustained silencing of miR-208a in the heart.^6^ Therapeutic inhibition of miR-208a by subcutaneous delivery of anti-miR-208a during hypertension-induced heart failure in Dahl hypertensive rats dose dependently prevents pathological myosin switching and cardiac remodeling, while improving cardiac function, overall health, and survival.^6^ These data recapitulated the cardioprotective effects seen after genetic deletion of miR-208a in mice.^7^

An intriguing feature of miRNA biology has been the minimal effects of miRNA loss of function under homeostatic conditions.^8^, ^9^ Instead, the actions of miRNAs in general seem to become pronounced under conditions of injury or stress. Thus, elimination of some miRNAs sensitizes cells to stress, resulting in exacerbated pathology, while the absence of other miRNAs can confer resistance to stress.^2^ While most anti-miR studies appear to indicate the absence of an effect under baseline, unstressed conditions, it is currently unknown whether disease (stress) influences the pharmacological effects of an anti-miR.

Using miR-208a as a model system, we show that stress changes the effect of anti-miR treatment on mRNA targets. In vitro analysis indicates that the influence of stress on anti-miR efficacy might depend on an increase in cellular anti-miR uptake under stress conditions. Together our data show that anti-miRs have stronger pharmacological effects during disease and that the origin of disease determines the therapeutic outcome. These considerations will be important for assessing the therapeutic dose and predicting the therapeutic effect in patients.

## Results

### The In Vivo Effect of Anti-miR-208a Is More Pronounced under Stress Conditions

**Figure 1:**
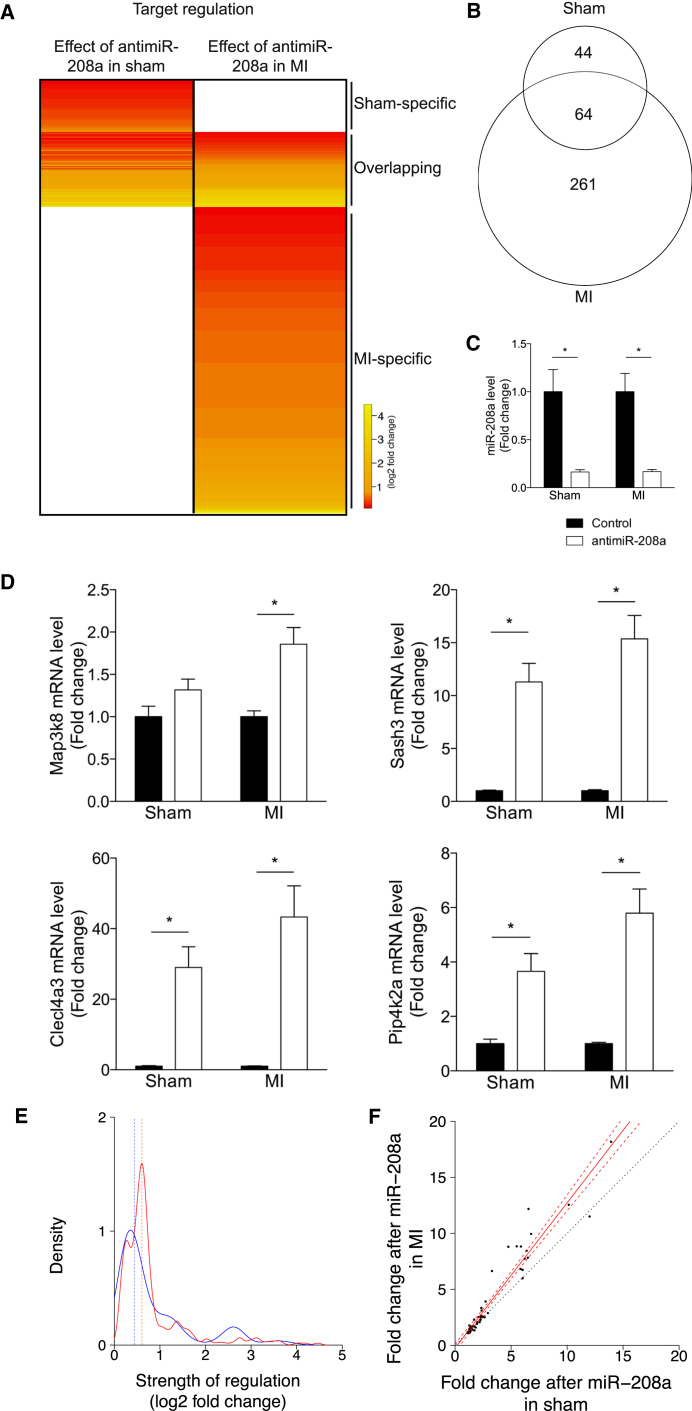
Target Derepression Is More Pronounced under Stress Conditions (A) Gene array analysis of LV tissue of rats that were subjected to sham operation (sham) or myocardial infarction (MI), after which both groups were treated with either control or anti-miR-208a. The heatmap expresses the average log2 fold change in expression for the significantly regulated miR-208a targets in anti-miR-208a-treated rats compared with control rats in either sham or MI rats (n = 4 per group). (B) Venn diagram showing the number of miR-208a targets that are significantly upregulated by anti-miR-208a in either the sham or MI rats. (C) Real-time PCR analysis of miR-208a showing inhibition after anti-miR-208a treatment. (D) Real-time PCR analysis of miR-208a targets shown to be upregulated by gene array after anti-miR-208a treatment in both sham and MI-operated rats. Data are expressed as mean fold change ± SEM and shown as fold change for sham anti-miR-208a (n = 6) over sham control (n = 6), and MI anti-miR-208a (n = 16–17) over MI control (n = 18–19). (E) Kernel density plot of the level of derepression (log2 fold change) of all upregulated targets after anti-miR-208a therapy in sham (blue line) or MI (red line) rats. (F) Fold change in miR-208a targets regulated after anti-miR-208a treatment in both sham rats (x axis) and MI rats (y axis). Solid red line is the linear regression, dashed red lines delineate the 99% confidence interval around the linear regression, and the dotted black line represents the identity line. *p < 0.05 for anti-miR-208a treatment versus control treatment.

AntimiRs function through the inhibition of a specific miRNA, and thereby have a derepressive effect on the direct targets of this miRNA. In an effort to explore whether the effect of anti-miR-208a changes under disease conditions, we performed microarray analysis on cardiac tissue from rats treated with either anti-miR-208a or control, and searched for mRNAs with a binding site for miR-208 that were significantly upregulated in the anti-miR-208a-treated group. This was done on left ventricular tissue in rats 8 weeks after they were subjected to sham surgery (sham) or myocardial infarction (MI) (n = 4 per group). Microarray analysis in the sham rats indicated that anti-miR-208a treatment resulted in the derepression of 108 genes that contained a potential miR-208a binding site in their 3′ untranslated region compared with control. However, while using the same treatment regimen, in the MI group inhibition of miR-208a resulted in the derepression of 325 genes that contain a potential miR-208a binding site in their 3′ untranslated region. Although 64 of these anti-miR-208a-regulated targets were overlapping in both the sham- and MI-operated rats, a large portion also appeared to be either sham- or MI-specific (Figures 1Aand 1B; Table S1).

To explore these data in more detail, we set out to determine the cardiac level of miR-208a in response to stress and after anti-miR-208a treatment. The levels of miR-208a did not change significantly in response to stress (Figure S1). Compared with control, miR-208a was significantly inhibited after anti-miR-208a treatment, with no detectable difference in the level of inhibition between sham and MI (Figure 1C). Real-time PCR analysis for a subset of randomly selected overlapping targets could largely confirm the upregulation of the genes after anti-miR-208a treatment compared with control in both sham and MI (Figure 1D; Figure S2A). This effect was not due to an effect of stress on the expression level of the mRNA targets, because these remained unchanged (Figure S2B).

Based on our microarray and the real-time PCR data, there appeared to be a trend toward a stronger derepression after MI compared with sham (Figures 1A and 1D). The level of derepression after anti-miR-208a treatment varied between 1.1- and 13.9-fold change for target genes regulated in the sham group, while this regulation was between 1.1 and 22.1 for the target genes regulated in the MI group. From the array data, we generated kernel density plots for the strength of derepression of the potential targets for both groups (n = 108 and n = 325) and observed a shift to the right for the strength of regulation in the MI group compared with sham, indicating that target derepression was generally stronger in MI than in sham (Figure 1E). We could confirm this observation when comparing the fold change in regulation in the subset of targets regulated in both the sham and the MI groups (n = 64) (Figure 1F).

Together, these data indicate that treatment with anti-miR-208a leads to derepression of more miR-208a targets after MI than after sham surgery and that the level of derepression is increased during disease.

### Target Regulation Can Be Dependent on the Type of Stress

**Figure 2:**
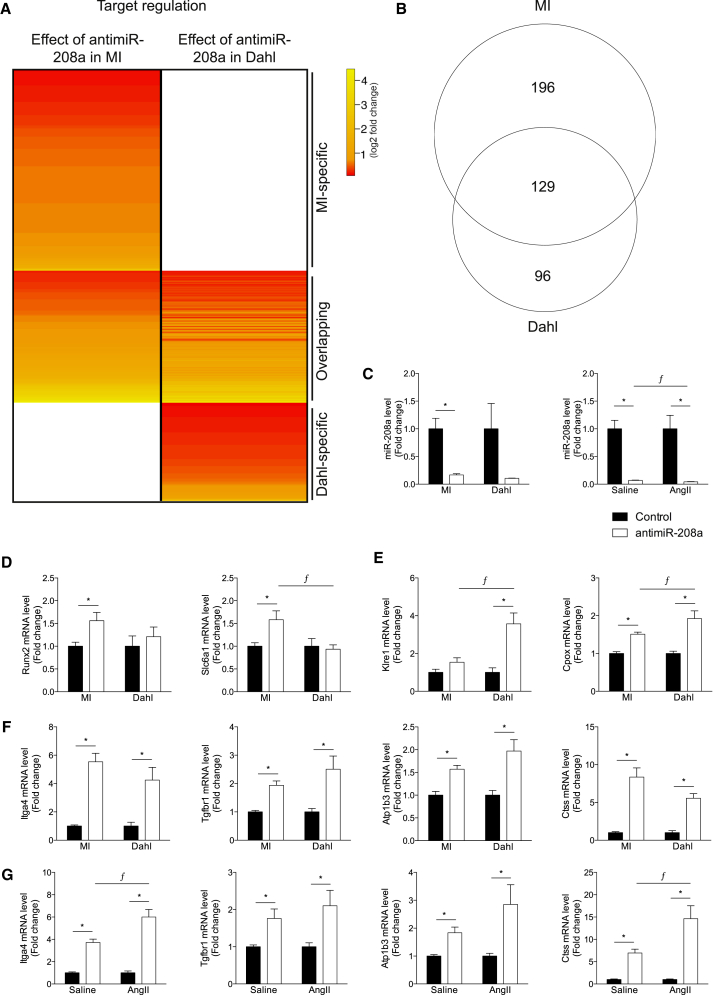
Target Derepression Is Dependent on the Type of Stress (A) Gene array analysis of LV tissue of rats that were subjected to myocardial infarction (MI) or Dahl rats on a high-salt diet, after which both groups were treated with either control or anti-miR-208a. The heatmap expresses the log2 fold change in expression for the significantly upregulated miR-208a targets in anti-miR-208a-treated rats compared with control rats in either MI or Dahl. (B) Venn diagram showing the number of miR-208a targets that are significantly upregulated by anti-miR-208a in either the MI or Dahl rats. (C) Real-time PCR analysis of miR-208a showing inhibition after anti-miR-208a treatment. (D) Real-time PCR analysis of miR-208a targets shown to be upregulated by gene array after anti-miR-208a treatment in MI rats, but not in Dahl rats. (E) Real-time PCR analysis of miR-208a targets shown to be upregulated by gene array after anti-miR-208a treatment in Dahl rats, but not in MI rats. (F) Real-time PCR analysis of miR-208a targets shown to be upregulated by gene array after anti-miR-208a treatment in both MI and Dahl rats. (G) Real-time PCR analysis of miR-208a targets in rats infused with angiotensin II (AngII) or vehicle (saline) and treated with anti-miR-208a or control. (D–G) The data are shown as mean fold change ± SEM and expressed as fold change for MI anti-miR-208a (n = 16–17) over MI control (n = 18–19), Dahl anti-miR-208a (n = 6–7) over Dahl control (n = 5–6), saline-infused anti-miR-208a (n = 5–6) over saline-infused control (n = 5–6), or AngII-infused anti-miR-208a (n = 5–6) over AngII-infused control (n = 6). *p < 0.05 for anti-miR-208a treatment versus control treatment, ^ƒ^p < 0.05 for anti-miR-208a treatment between models.

To determine whether the type of stress also influences the targets that are regulated by an miRNA, we compared the effect of anti-miR-208a during cardiac remodeling in response to MI with the effect of anti-miR-208a in Dahl salt-sensitive rats on a high-salt diet.^10^ Dahl rats develop hypertension and subsequent cardiac remodeling in response to a high-salt diet.^10^ Compared with the 325 targets regulated in response to anti-miR-208a after MI, we found 225 targets to be derepressed in the Dahl rats with a derepression level that varied between 1.1- and 18.3-fold change compared with control (Figures 2A and 2B; Table S2).

Real-time PCR analysis on cardiac tissue after anti-miR-208a treatment showed a profound inhibition of miR-208a for both MI and Dahl hearts (Figure 2C). Of all the 325 regulated targets, 196 targets were specific for rats exposed to MI. Real-time PCR analysis for randomly selected MI-specific target genes confirmed the MI-specific derepression by anti-miR-208a (Figure 2D; Figure S3). Also, for the Dahl-specific targets we could validate the derepression by real-time PCR, showing derepression after anti-miR-208a compared with control (Figure 2E; Figure S4). Although microarray analysis indicated these targets to be either MI or Dahl specific, real-time PCR analysis actually showed that some targets were also regulated after anti-miR-208a treatment in the other stress group (Figures 2D and 2E; Figures S3A and S4A). This observation might be because of the greater sensitivity and the amplification steps of the real-time PCR assay compared with the microarray analysis or the larger number of animals used in the real-time analyses.

The random selection of overlapping targets showed a strong derepression after anti-miR-208a compared with control (Figure 2F; Figures S5A and S5B), indicating that these targets are regulated by miR-208a, independent of the cause of disease. Indeed, also in angiotensin II (AngII)-infused rats,^11^ another rat model to induce cardiac remodeling, these targets were confirmed to be derepressed after anti-miR-208a treatment compared with control (Figure 2G; Figure S6). Although to a lower extent, the derepression of these selected targets also reached significance in the unstressed (saline) groups treated with anti-miR-208a (Figure 2G; Figure S6), while miR-208a inhibition appeared comparable (Figure 2C). Stress by itself appears to influence some of the targets (Figures S3B, S4B, S5B, and S6B).

These data show that a large portion of targets is consistently regulated across different disease models upon anti-miR-208a treatment. However, the fact that we can observe derepression for different genes depending on the cause of disease implies that miR-208a also regulates a divergent set of gene targets dependent on the disease etiology.

### Anti-miR Efficacy Is Dependent on the Level of Stress

**Figure 3:**
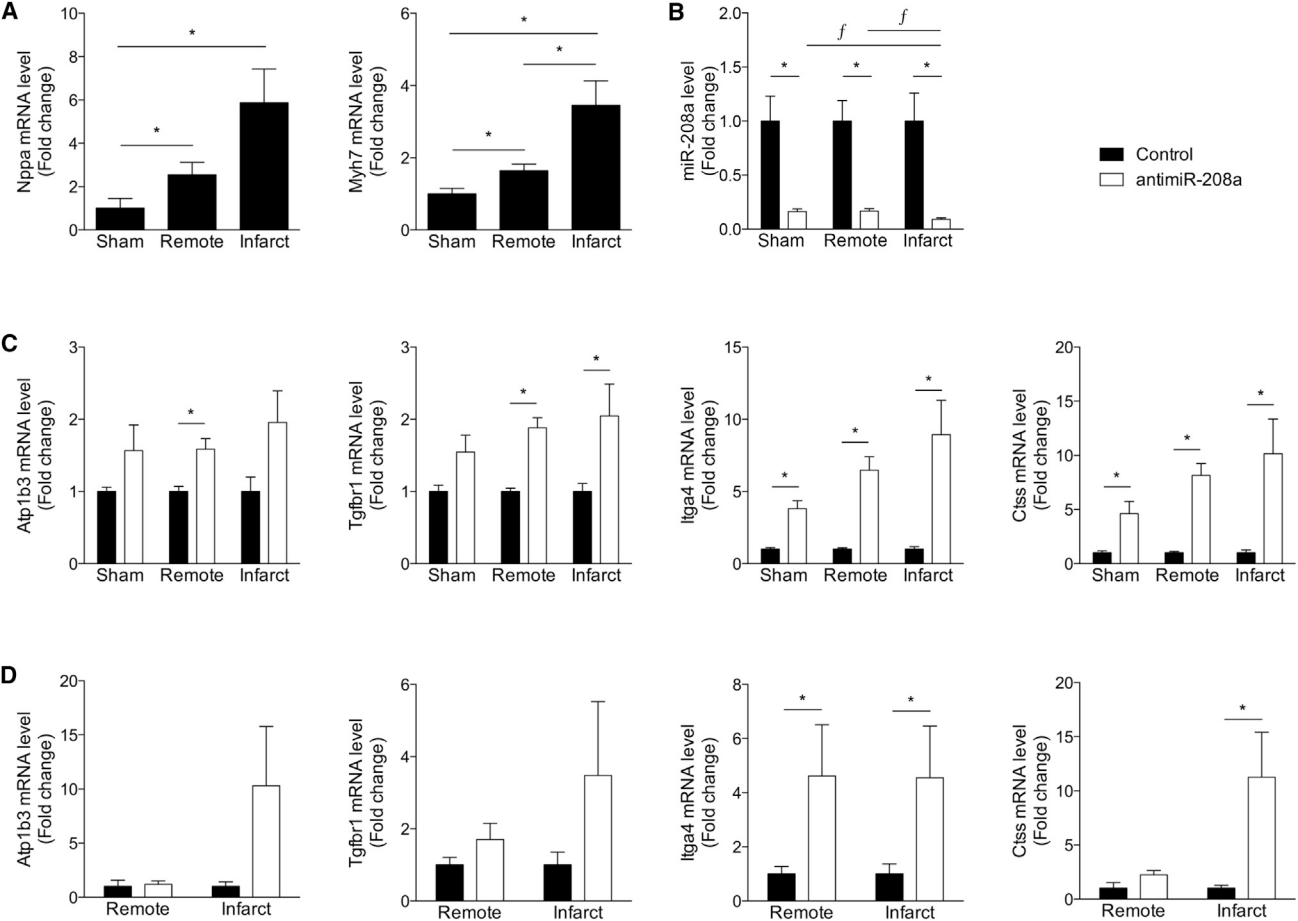
Anti-miR Efficacy Depends on the Level of Stress (A–C) Real-time PCR analysis of cardiac stress markers (A), miR-208a levels (B), or miR-208a target genes (C) on LV tissue from sham-operated rats (sham) or different regions of MI-operated rats (remote, infarct) after control or anti-miR-208a treatment. (D) Real-time PCR analysis of miR-208a target genes on LV tissue from different regions of infarcted pig hearts (remote, infarct) after control or anti-miR-208a treatment. Data are shown as mean fold change ± SEM and expressed as fold change for sham anti-miR-208a (n = 6–7) over sham control (n = 5–6), MI remote anti-miR-208a (n = 15–17) over MI remote control (n = 17–19), MI infarct anti-miR-208a (n = 15–17) over MI infarct control (n = 17–19) or pig IR remote anti-miR-208a (n = 3–4) over pig IR remote control (n = 6–7), and pig IR infarct anti-miR-208a (n = 3–4) over pig IR infarct control (n = 5–6). *p < 0.05 for anti-miR-208a treatment versus control treatment; ƒ p < 0.05 for infarct or remote compared to sham.

Next, we aimed to investigate whether the severity of stress also influences the level of target derepression in response to anti-miR treatment. To this end, we collected tissue from sham hearts and both the remote and the infarct region after MI. Because an infarct induces a local injury to cardiac tissue, the area surrounding the damaged area presumably experiences more stress than more remote tissue. Indeed, the expression of both *Nppa* and *Myh7*, both well-known markers for cardiac stress,^12^, ^13^ were more highly expressed moving from the remote toward the infarcted region, indicating a gradient of stress exposure in these different regions (Figure 3A). Real-time PCR analysis of the level of miR-208a shows a profound miR-208a repression in cardiac tissue of sham rats and the remote and infarct regions of MI rats in response to anti-miR-208a treatment. Additionally, the miR-208a reduction in the infarct region was significantly bigger compared with the remote region or sham hearts (Figure 3B). Although it did not reach statistical significance, the strength of regulation of the analyzed targets appeared to be trending to increase with an increasing level of stress (Figure 3C; Figure S7). A comparable analysis in cardiac tissue from pig hearts exposed to ischemia-reperfusion (IR) indicated that the miR-208a targets were conserved across species and the trend in larger derepression in the infarcted compared with the remote region was also observed in larger animals (Figure 3D; Figure S8).

Together, these data suggest that the level of stress influences the level of target derepression after anti-miR-208a treatment, and that target derepression for this subset of these genes is conserved in a large animal model of MI.

### Cellular Uptake of Anti-miR Changes under Stress Conditions

**Figure 4:**
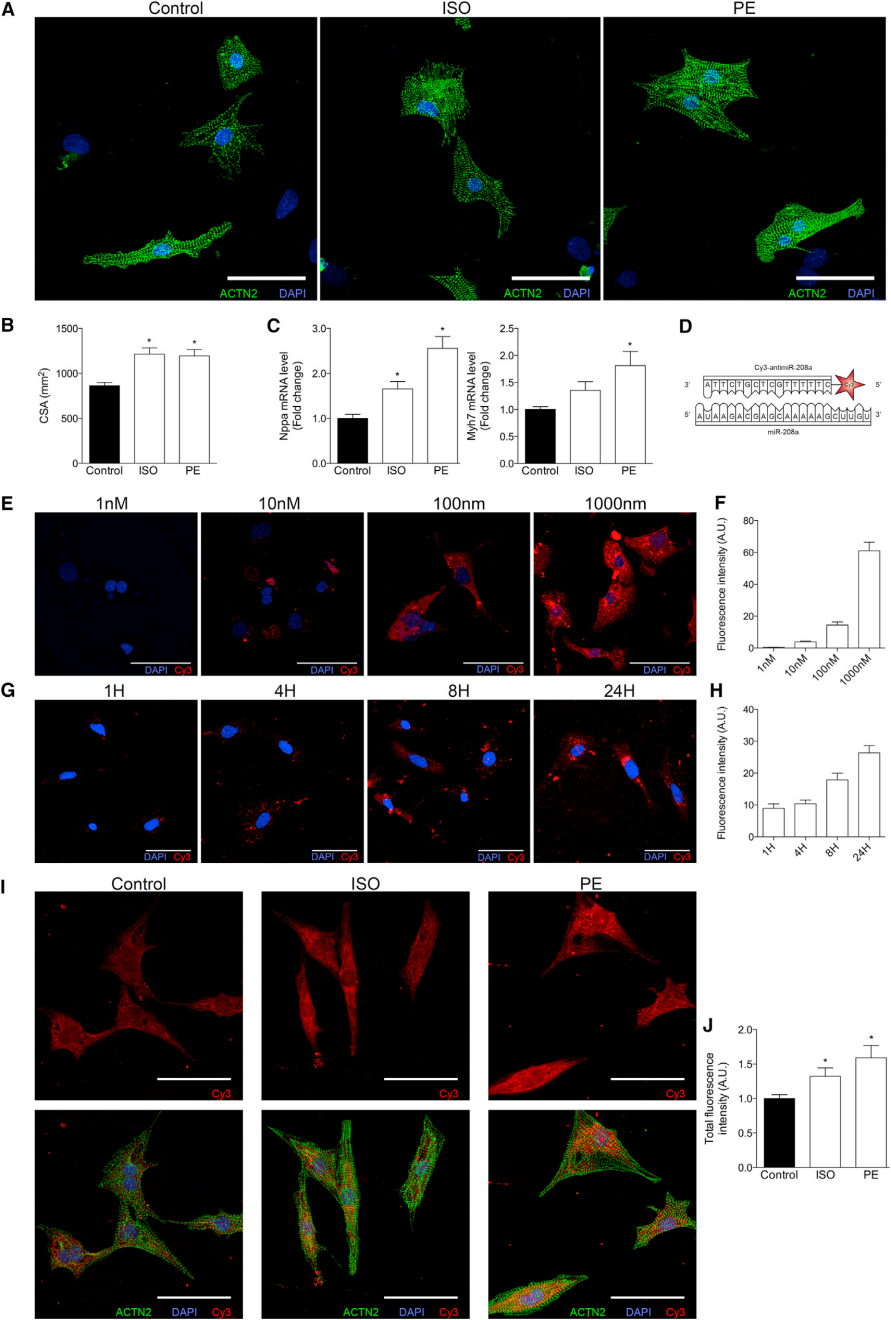
Stress Influences Cellular Uptake of Anti-miRs in Neonatal Rat Ventricular Myocytes (A) NRVMs stained for ACTN2 after treatment with or without isoproterenol or phenylephrine for 24 hr. (B) Quantification of cross-sectional area (CSA) of NRVMs in the presence or absence of ISO or PE (per condition, five to six biological samples were generated, and per samples, ±22–41 cells were quantified). (C) Real-time PCR analysis of stress marker expression in NRVMs in the presence or absence of ISO or PE (n = 6 per condition). (D) Schematic representation of miR-208a and the Cy3-labeled anti-miR-208a. (E) Fluorescent images of NRVMs treated with increasing doses of Cy3-anti-miR-208a for 24 hr. (F) Quantification of Cy3 signal at different concentrations of anti-miR-208a. (G) Fluorescent images of NRVMs treated with 1 μM Cy3-anti-miR-208a for different time periods. (H) Quantification of Cy3 signal at different time points after 1 μM Cy3-anti-miR-208a. (I) Fluorescent images of NRVMs that were either left untreated or stimulated with ISO or PE for 8 hr, after which they received 1 μM Cy3-anti-miR-208a. (J) Quantification of total fluorescence (fluorescence intensity corrected for cell size, n = 8–12). Data are represented as mean fold change ± SEM. *p < 0.05 for anti-miR-208a treatment versus control treatment. Scale bars represent 50 μM.

In an effort to explore the mechanism behind the increased target derepression under stress conditions, we used neonatal rat ventricular myocytes (NRVMs) exposed to isoproterenol (ISO) or phenylephrine (PE),^5^ both known inducers of cardiomyocyte hypertrophy (stress).^14^ Cell size quantification confirmed the presence of cardiomyocyte hypertrophy in response to both ISO and PE (Figures 4A and 4B). The increase in cell size corresponded to an increase in the expression of the cardiac stress markers Nppa and Myh7, indicating cardiomyocyte stress (Figure 4C). To be able to track anti-miR-208a in vitro, we treated NRVMs with a Cy3-labeled anti-miR-208a (Figure 4D). To replicate in vivo therapy as best we could, no transfectants were used to aid uptake of the anti-miR. Fluorescence intensity of individual cells was used as a measure of uptake of the labeled anti-miR. Fluorescence intensity increased upon treatment with increasing doses (Figures 4E and 4F) or increased incubation time (Figures 4G and 4H). Although uptake was detected in unstressed cardiomyocytes, the cells appeared to take up more anti-miR under conditions of stress (Figure 4I) Quantification of uptake by measuring total cellular fluorescence revealed a significantly increased uptake in response to both stresses (Figure 4J). These data imply that an increase in cellular uptake with stress might be partially responsible for an increase in target derepression under disease conditions.

## Discussion

AntimiRs have shown to be efficacious in establishing therapeutic benefit under multiple disease conditions. Mouse genetics has shown us that miRNA functions are often more pronounced during disease and that the regulated mRNA targets can depend on the disease condition.^15^ Also, oligo-based microRNA inhibition has shown comparable effects. Independent of the anti-miR chemistry used (either antagomirs or locked nucleic acid [LNA]-modified anti-miRs) or tissue studied, several cases have been reported where there was a larger effect on specific target depression under conditions of stress.^16^, ^17^, ^18^ However, so far, these observations have remained unstudied, and it is unknown whether the effect of stress on anti-miR efficacy is a common phenomenon and/or whether the level and type of stress influences anti-miR function. To answer these research questions in vivo, we used an anti-miR, specifically targeting miR-208a. The cardiomyocyte-restricted expression of miR-208a prevents any interfering effects of cellular differences in target regulation, providing a clean experimental model to study the effect on target regulation by anti-miR-208a.

Our data indicate that anti-miR treatment under diseased conditions results in derepression of a larger number of target genes than anti-miR treatment under baseline conditions. Additionally, we show that the strength of target regulation is increased during stress in rats, which we can confirm in a porcine model of ischemic injury.

There are many potential explanations for our observations. The impact of stress on anti-miR efficacy might be because of a change in anti-miR activity or a direct effect of stress on target regulation. Stress might increase miR-208a inhibition by an increase in cellular uptake of anti-miR-208a, endosomal escape of anti-miR-208a, a change in cellular localization of either the anti-miR or miRNA that changes their interaction, or even an expressional change in long non-coding RNAs (lncRNAs) during stress that might influence the effect of the anti-miR by changing the cellular level of miR-208a.

The effect of an miRNA on its target depends on the ratio of miRNA to target^19^ and is cell and context dependent.^20^ Both a change in mRNA transcripts available to target by the miRNA as well as a change in miRNA activity could be responsible for the increased number of genes that are regulated during disease.^21^ Disease might trigger an increase in expression of miRNA target genes, which thereby become susceptible to and available for miRNA regulation. In parallel it might also be caused by a change in miRNA function (abundance or activity) that is directing the increased number of gene targets that are being regulated during disease. Other possible explanations are that the efficiency of target regulation improves because of a change in secondary structure of the target region, a change in efficiency of RNA-induced silencing complex (RISC) loading, or an effect of stress on the cellular localization of either the miRNA or the mRNA. Obviously it could also very well be a combination of these proposed mechanisms that might additionally be miRNA or target dependent. Although these aspects deserve further investigation, we know that miR-208a levels, because it is co-expressed with Myh6, remain unchanged or even decrease under conditions of disease, so miR-208a abundance is not the explanation in this particular study.

Although the anti-miRs are highly specific in targeting an miRNA, we can currently not exclude that the difference in target regulation is also partly due to a change in abundance of additional miRNAs that can also regulate the measured transcripts.

Although the level of target regulation is likely also under the influence of mRNA abundance and activity of the miRNA itself, our in vitro data show that the stronger effect of anti-miR treatment under diseased conditions could potentially also be caused by an enhanced uptake of the compounds when the cells become stressed. Several attempts to confirm this in vivo by injecting labeled anti-miR-208a failed, likely because of inefficient targeting of the heart.

Our study also showed that the genes that are derepressed by an anti-miR partially depend on the disease driver. This effect is probably due to the fact that the target miRNA is regulating a different gene set when divergent signaling pathways and genes are activated in response to different stressors. Hypertension-induced cardiac remodeling will activate a different gene program than cardiac remodeling in response to MI. Therefore, it seems fair to assume that the divergence in gene regulation caused by the presence of stress or under different disease conditions is largely due to the availability of the mRNA targets present in the cells.

It is necessary to measure target derepression to demonstrate efficacy of target engagement by anti-miRs in vivo. Although miRNA-induced changes in gene expression can occur at both the mRNA and the protein levels, the majority of changes occur because of mRNA destabilization.^22^ The average level of target regulation is normally modest and ranges between 20% and 50% change in mRNA, making it difficult to determine significant changes above naturally occurring variation in gene expression.^23^, ^24^ Additionally, proteomic studies in response to miRNA modulation have reported that the average changes in protein levels of miRNA targets are less than 2-fold following miRNA inhibition.^25^, ^26^ Although our microarray data confirmed this level of regulation for the majority of the targets, our real-time PCR data indicated a greater fold change after anti-miR-208a treatment. It is currently unknown whether this increase in fold regulation is introduced by our experimental setup. Nonetheless, real-time PCR analysis did confirm the derepression of most targets identified by microarray.

Although the exact mechanisms of enhanced target regulation under diseased conditions remain to be defined, our observations could have far-reaching implications for the clinical use of anti-miRs as novel therapeutics. The stronger pharmacological effects of anti-miRs during disease and the fact that disease etiology determines the therapeutic outcome of an anti-miR will be important for assessing the therapeutic dose and predicting the therapeutic effect in patients. Although this is important information to take along in developing an optimal therapy, both the importance of miRNAs and the potency of anti-miRs support enthusiasm for further pursuing these gene expression regulators as novel therapeutic candidates.

## Materials and Methods

### Animal Studies

Animal experiments were performed in accordance with the Institutional Animal Care and Use Committee (IACUC) at miRagen Therapeutics (anti-miR studies in sham- or MI-operated rats and high-salt diet Dahl rats), the institutional review committee of the Royal Netherlands Academy of Arts and Sciences (KNAW) (anti-miR studies in angiotensin II-treated rats), or the Servier Research Institute ethical committee (porcine studies), and they comply with the federal and state guidelines concerning the use of animals and research as defined by the Guide for the Care and Use of Laboratory Animals (NIH Publication No. 80-23, revised 1985) or with national animal welfare laws under a project license of the Dutch or French government.

#### Rat MI Studies

Adult Wistar (Charles River) male rats were anesthetized with isoflurane at 5% for 2–3 min, intubated, and ventilated using a rodent ventilator (Hallowell Microvent I). Isoflurane was maintained at 1.5%–2.5%. Surgery was performed on a heated plate to maintain body temperature at 37°C; body temperature was monitored via rectal probe. The heart was reached through a left-sided thoracotomy between ribs 4 and 5; the left anterior coronary artery was ligated using a 7-0 silk suture. Successful ligation was confirmed by loss of color of the myocardium distal to the ligation. The chest wall and skin were closed, and rats were allowed to recover. Once awake, they were removed from ventilator and moved to a warm recovery cage. Once fully ambulatory, they were returned to normal housing and given 0.01 mg/kg buprenorphine subcutaneously. Sham-operated rats were subjected to the same procedure, with the exception that no ligation of the coronary artery was performed. After surgery the rats were kept for 8 weeks before sacrifice and tissue collection.

#### Dahl Salt-Sensitive Rat

Male Dahl rats (Harlan) were maintained on a 6% NaCl diet for 8 weeks starting at 7 weeks of age.

#### Angiotensin II Delivery

Adult male Sprague-Dawley rats received angiotensin II (AngII; 0.25 mg/kg/day; Sigma-Aldrich) for 8 weeks by osmotic minipumps (ALZET model 2004; DURECT Corporation). Rats were anesthetized with 4%–5% isoflurane and maintained with 1%–2% isoflurane supplemented with oxygen. Surgery was performed on a heated plate to maintain body temperature at 37°C. A subcutaneous pocket on the back of the rats (interscapular) was created using blunt-end scissors, after which the AngII-filled osmotic minipump was placed in this pocket and the wound was closed with wound clips (ALZET; DURECT Corporation). After 4 weeks the pump was replaced with a new AngII-filled pump. Control animals received the same procedure with pumps filled with vehicle (saline).

#### Porcine IR Studies

Göttingen minipigs (Ellegaard) were subjected to ischemia-reperfusion (IR) by a closed-chest approach and clinical cardiac catheterization techniques.^27^, ^28^ Adult (12–15 months) male pigs were sedated with a mixture of tiletamine and zolazepam (Zoletil; 15 mg/kg). Following adequate sedation, the neck was shaved, properly scrubbed, and disinfected with Vetedine soap (Vetoquinol). An intravenous catheter was placed in a marginal vein of one ear for the administration of fluids and anesthetic agent, an endotracheal tube was inserted for mechanical ventilation, and body temperature was maintained at 36.5°C–39°C with a blanket during the procedure. Surface electrocardiogram (ECG) was used to monitor the onset of arrhythmias (ventricular tachycardia and fibrillation). Thiopental was used (∼10 mg/kg/hr intravenously [i.v.]) for stable and prolonged anesthesia.

The carotid artery was exposed and access gained by the Seldinger technique. Under fluoroscopy guidance, a JR3.5 catheter was advanced over the wire to the level of the coronary sinus and placed at the coronary ostium without full engagement. Good placement was confirmed with a bolus of contrast agent (Telebrix) to visualize the left main, circumflex, and anterior descending coronary. Heparin (300 UI/kg i.v.) was given just before left anterior descending artery (LAD) occlusion to prevent clotting. A guide wire was advanced into the LAD followed by a balloon catheter (3.5 × 15–20) that subsequently was inflated at 4–6 atmosphere (atm) to ensure occlusion of the LAD for 150 min. After the 150 min occlusion, the balloon catheter was deflated and slowly removed. A bolus of lidocaine (2 mg/kg i.v.) and nitroglycerin (40 μg/kg intracoronary) was administered to avoid vasospasm and arrhythmia. During the recovery period, buprenorphine (50 μg/kg intramuscularly [i.m.]) was given for analgesia. After waking up under a warming lamp the pigs were extubated, and butorphanol (Dolorex; 0.2 mg/kg subcutaneously [s.c.]) and amoxicillin (Dufamox; 0.015 mg/kg i.m.) were given for post-procedural care.

### Anti-miR Injections

AntimiRs (designed and synthesized by miRagen) were dissolved in saline and delivered by subcutaneous injection. Used anti-miRs are LNA-DNA mixmers; these 16-mers are complementary to miR-208a specifically and have high nuclease resistance, as well as high duplex melting temperature.^6^ Animals were injected every other week with 25 mg/kg anti-miR-208a or a comparable volume of vehicle starting 1 week after the intervention (sham or MI, high-salt [HS] diet, PBS or AngII).

### RNA Extraction

Total RNA was extracted from left ventricular (LV) tissue or cultured NRVMs with TRIzol according to the manufacturer’s protocol (Invitrogen).

### Gene Expression Analysis

Gene expression profiling was performed by a service provider (MOgene) on Agilent SurePrint G3 Rat Gene Expression, 8 × 60 microarrays. Sample integrity was assessed with Agilent Bioanalyzer prior to microarray analysis. Data were analyzed using Array Studio software. Significantly regulated genes were defined using a Benjamini-Hochberg false discovery rate (BH-FDR) corrected p value cutoff of ≤0.05 to control for multiple testing. Differential gene expression reflects statistically significant expression in the treatment group compared with the saline-treated group. Hierarchical clustering was performed using the software program R using an agglomerative metric with Euclidean distance. For this study, genes containing a 6-, 7-, or 8-mer miR-208a binding site in their 3′ untranslated region were considered as potential miR-208a target. For these target genes, an average fold change in gene expression after anti-miR-208a treatment over control was calculated for each animal model (n = 4 per group). The genes that show a significant derepression after anti-miR-208a were used to generate the heatmaps shown in Figures 1A and 2A. A cutoff p value of <0.05 was used to determine differential expression.

### Real-Time PCR

cDNA was synthesized from 400–1,000 ng of total RNA extracted from tissue or cells using the iScript cDNA Synthesis Kit (Bio-Rad) for genes and miScript II RT Kit (QIAGEN) for miRNAs. Real-time PCR was performed to analyze the expression levels of individual mRNAs/miRNAs, using a specific set of primers (Table S3) and iQ SYBR Green Supermix (Bio-Rad) on a real-time PCR machine (CFX Connect Real-Time PCR Detection System; Bio-Rad). Expression levels were normalized to the levels of glyceraldehyde 3-phosphate dehydrogenase (GAPDH) mRNA levels for genes/RNU6b small nucleolar RNA (snRNA) (U6) for miRNAs, and fold changes in gene and/or miRNA expression were calculated according to the 2^−ΔΔCT^ method and expressed as mean fold change ± SEM.

### Cell Culture and Neonatal Rat Cardiomyocytes

Neonatal rat ventricular cardiomyocyte (NRVM) cultures were isolated by enzymatic dissociation of neonatal rat hearts, as described previously.^2^ In short, hearts from 1- to 2-day-old rat pups were collected, the atria were removed, and the ventricular cells were enzymatically dissociated with trypsin (Life Technologies) in a water (37°C) jacketed spinner flask. The single-cell suspension was filtered and pre-plated to remove debris and non-myocytes, respectively. Primary cardiomyocytes were initially maintained in Ham’s F10 medium (GIBCO) supplemented with 5% fetal bovine serum (FBS; Sigma-Aldrich) and 1% penicillin-streptomycin (Life Technologies). The day after isolation, cardiomyocytes were switched to serum-free Ham’s F10 medium, supplemented with 1% penicillin-streptomycin and 1 μl/ml insulin-transferrin-sodium-selenite supplement (catalog number 11074547001; Sigma-Aldrich).

For stress experiments, isoproterenol (ISO; 10 μM final concentration, catalog number I6504; Sigma-Aldrich) or phenylephrine (PE; 10 μM final concentration, catalog number P6126; Sigma-Aldrich) was added to the (serum-free) culturing medium. For subsequent anti-miR treatment, the Cy3-labeled anti-miR-208a (1 μM final concentration, unless otherwise indicated) was added to the medium after 8 hr of culture (passive delivery), without removing the stressor. Cells were then cultured for a further 16 hr before fixing and imaging.

### Confocal Microscopy

NRVMs cultured on coverslips were fixed with 4% paraformaldehyde, permeabilized with 1% Triton X-100 (Sigma-Aldrich), blocked with 1% fish gelatin (Sigma-Aldrich), and stained with an anti-α-actinin primary antibody (ACTN2; 1:500; A7811; Sigma-Aldrich) and an Alexa 488-conjugated secondary antibody (1:200; A11001; Sigma-Aldrich). Coverslips were then mounted with ProLong Gold antifade reagent with DAPI (P36935; Life Technologies). Cells were imaged using a Leica TCS SPE. Cross-sectional area (CSA) and uptake of Cy3-labeled anti-miR (as indicated by Cy3 fluorescence intensity) were measured using ImageJ. For all experiments where fluorescence intensity was quantified, laser and detector settings were kept consistent across the experiment. Quantifications were based on 17–43 cells per biological replicate, collected from 10 fields per replicate, for 8–12 replicates per condition.

### Statistical Analysis

Values are presented as mean ± SEM. Outliers were identified and excluded using a Grubbs’ test (using α = 0.05; GraphPad). Statistical significance was evaluated using an unpaired t test for comparisons between two groups, using GraphPad Prism software. The kernel density plot was generated using R, as were the regression line and matching confidence interval in Figure 1E. A p value <0.05 was considered significant.

## Author Contributions

Conceptualization, J.E.C.E., C.J.D., and E.v.R.; Formal Analysis, J.M.L., J.E.C.E., and C.J.D.; Investigation, J.E.C.E., C.J.D., A.G.S., R.L.M., H.M.S., A.L.J., M.I., and S.C.; Resources, A.G.S., R.L.M., H.M.S., A.L.J., M.I., and S.C.; Writing – Original Draft, J.E.C.E., C.J.D., and E.v.R.; Writing – Review & Editing, J.E.C.E., C.J.D., and E.v.R.; Visualization, J.E.C.E. and C.J.D.; Supervision, E.v.R.; Funding Acquisition, E.v.R.

## Conflicts of Interest

J.M.L., A.G.S., R.L.M., H.M.S., and A.L.J. are employees of miRagen Therapeutics. M.I. and S.C. are employees of Servier. E.v.R. is a scientific co-founder and member of the Scientific Advisory Board of miRagen Therapeutics, Inc.

## Supplemental Information

Document S1. Figures S1–S8 and Table S3

Table S1. Fold Change of Seed-Matched miR-208a Target Genes that Are Significantly Upregulated in Response to Anti-miR-208a Treatment after Sham or MI Surgery

Table S2. Fold Change of Seed-Matched miR-208a Target Genes that Are Significantly Upregulated in Response to Anti-miR-208a Treatment after MI Surgery or in High-Salt-Diet-Fed Dahl Rats

Document S2. Article plus Supplemental Information
