# Supplementary material for: The Efficacy of Cardiac Anti-miR-208a Therapy Is Stress Dependent
Source: Mol Ther. 2017 Feb 12;25(3):694–704. doi: 10.1016/j.ymthe.2017.01.012 (PMC5363185; doi:10.1016/j.ymthe.2017.01.012)
Supplement: Document S1. Figures S1–S8 and Table S3 [file mmc1.pdf]

YMTHE, Volume *25*

Supplemental Information

The Efficacy of Cardiac Anti-miR-208a Therapy Is Stress Dependent

Joep E.C. Eding, Charlotte J. Demkes, Joshua M. Lynch, Anita G. Seto, Rusty L. Montgomery, Hillary M. Semus, Aimee L. Jackson, Marc Isabelle, Stefano Chimenti, and Eva van Rooij

Figure S1

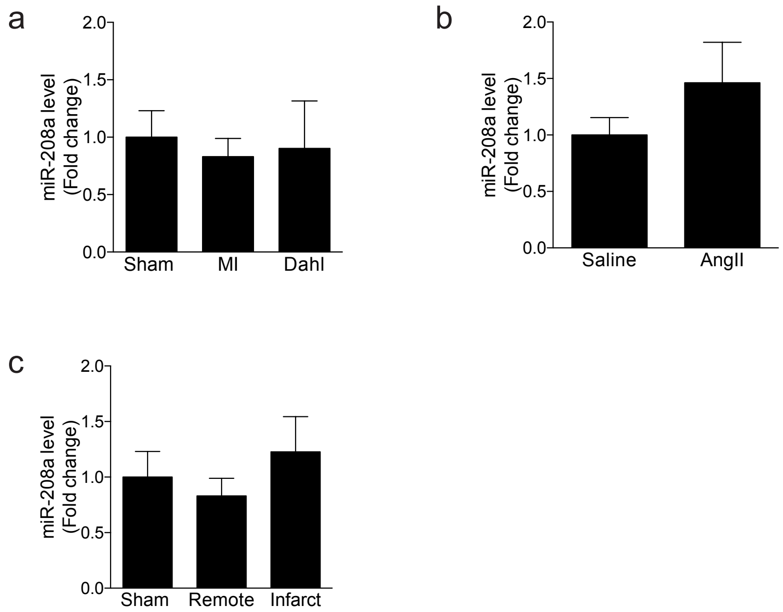

Figure S1. Real-time PCR analysis for miR-208a on LV tissue in **(a)** sham rats, MI rats and Dahl rats, **(b)** saline- and AngII-infused rats, **(c)** sham-operated rats (Sham) or different regions of MI-operated rats (Remote, Infarct). Data are expressed as mean fold change ± SEM for Sham control (n=6), MI control (n=18), Dahl control (n=6), saline control (n=6), AngII control (n=6), MI remote control (n=18) and MI infarct control (n=19).

Figure S2

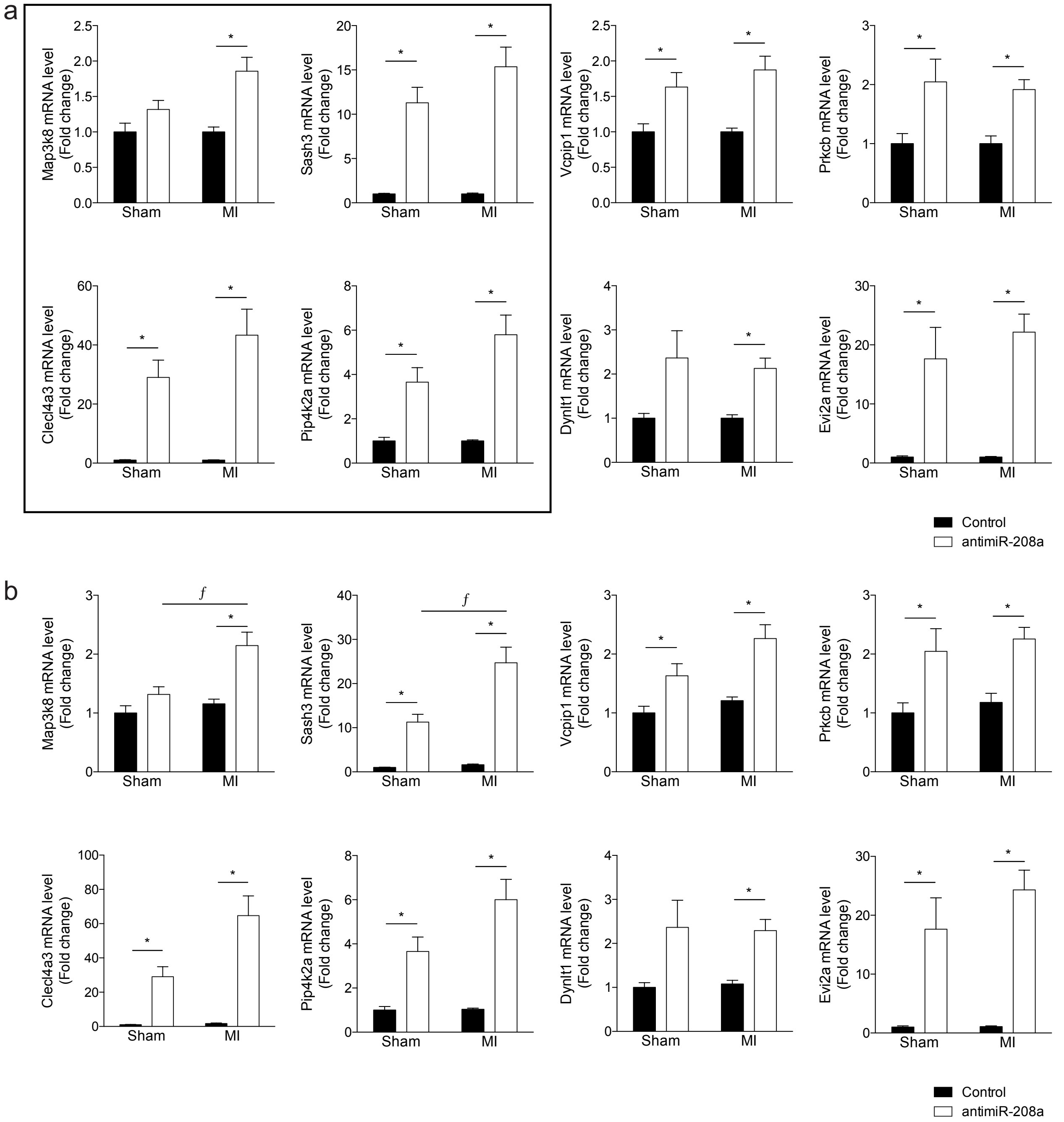

Figure S2. Real-time PCR analysis of miR-208a targets shown to be upregulated by gene array after antimiR-208a treatment in both MI and sham surgery. (**a**) Data are expressed as mean fold change ± SEM for Sham antimiR-208a (n=6) over Sham control (n=6) and MI antimiR-208a (n=16-17) over MI control (n=18-19). (**b**) Data are expressed as mean fold change ± SEM for Sham antimiR-208a (n=6), MI control (n=18-19) and MI antimiR-208a (n=16-17) over Sham control (n=6). * indicates $p<0.05$ for antimiR-208a treatment versus control treatment; *f* indicates $p<0.05$ for antimiR-208a treatment between models. Boxed graphs are shown in main figure 1d.

Figure S3

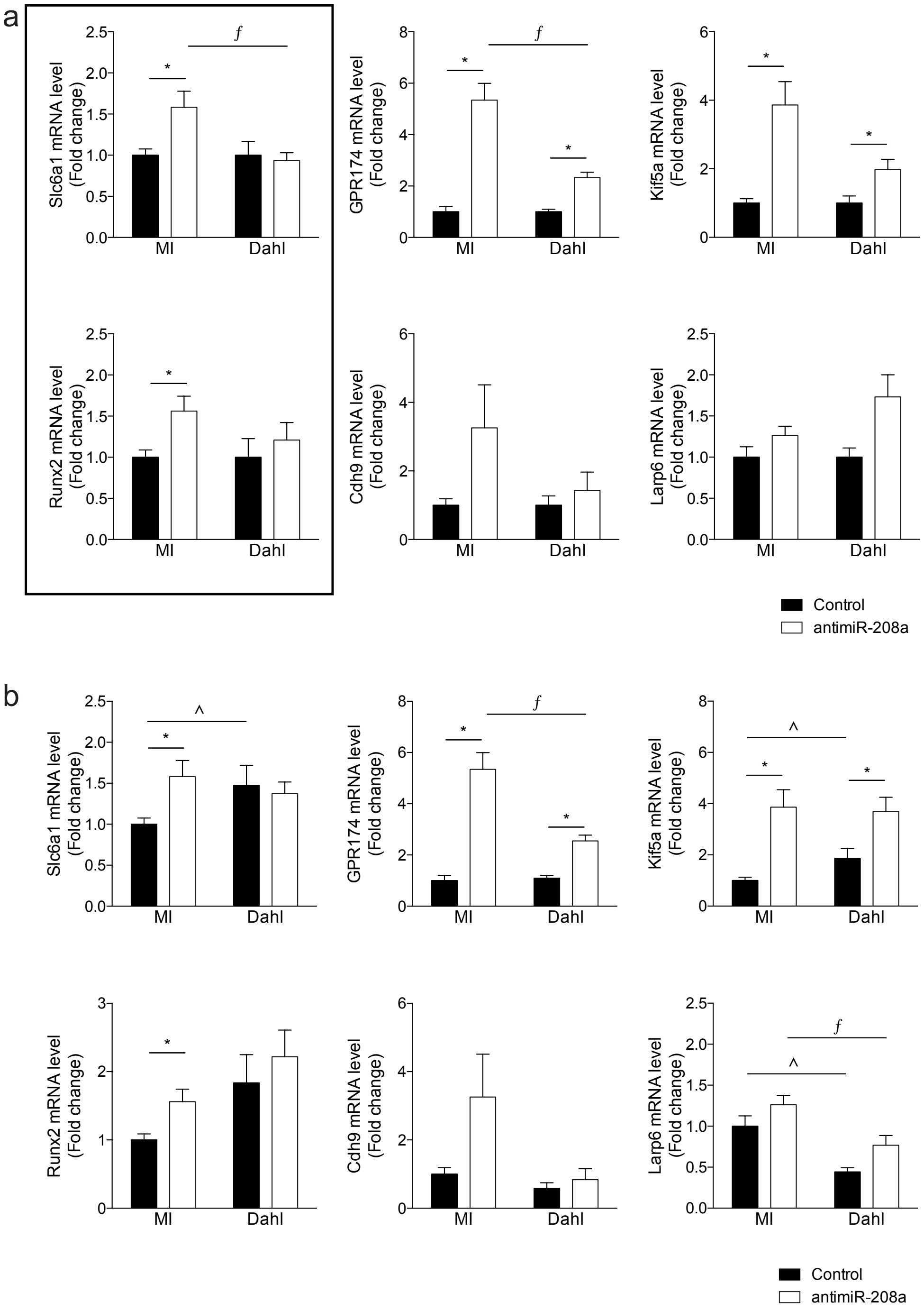

Figure S3. Real-time PCR analysis of miR-208a targets shown to be upregulated by gene array after antimiR-208a treatment after MI surgery but not in Dahl rats. (**a**) Data are expressed as mean fold change ± SEM for MI antimiR-208a (n=16-17) over MI control (n=18-19) and Dahl antimiR-208a (n=6-7) over Dahl control (n=5-6). (**b**) Data are expressed as mean fold change ± SEM for MI antimiR-208a (n=16-17), Dahl control (n=5-6) and Dahl antimiR-208a (n=6-7) over MI control (n=18-19). * indicates $p<0.05$ for antimiR-208a treatment versus control treatment; ^ indicates $p<0.05$ for control treatment between models; ƒ indicates $p<0.05$ for antimiR-208a treatment between models. Boxed graphs are shown in main figure 2d.

Figure S4

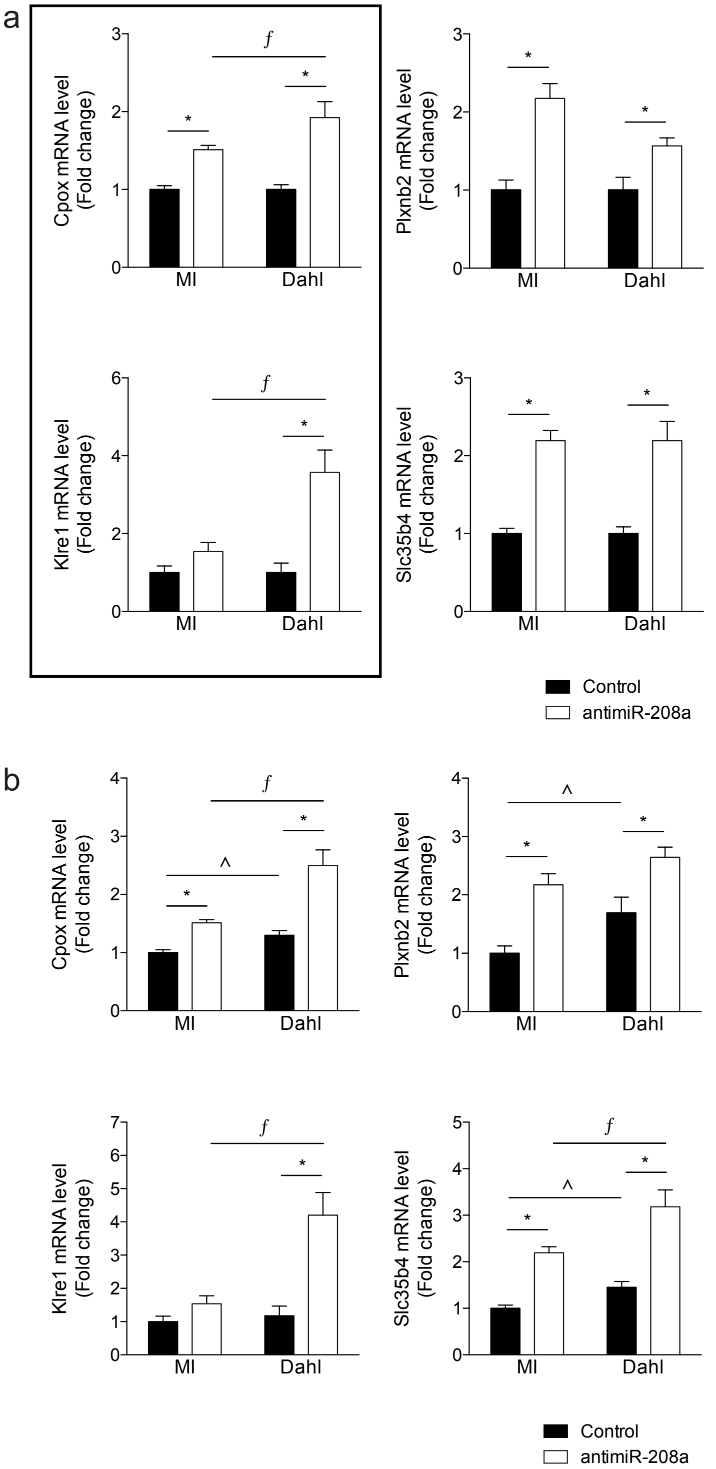

Figure S4. Real-time PCR analysis of miR-208a targets shown to be upregulated by gene array after antimiR-208a treatment in Dahl rats, but not after MI surgery. (**a**) Data are expressed as mean fold change ± SEM for MI antimiR-208a (n=15) over MI control (n=16-19) and Dahl antimiR-208a (n=6-7) over Dahl control (n=5-6). (**b**) Data are expressed as mean fold change ± SEM for MI antimiR-208a (n=15), Dahl control (n=5-6) and Dahl antimiR-208a (n=6-7) over MI control (n=16-19). * indicates $p<0.05$ for antimiR-208a treatment versus control treatment; ^ indicates $p<0.05$ for control treatment between models; *f* indicates $p<0.05$ for antimiR-208a treatment between models. Boxed graphs are shown in main figure 2e.

Figure S5

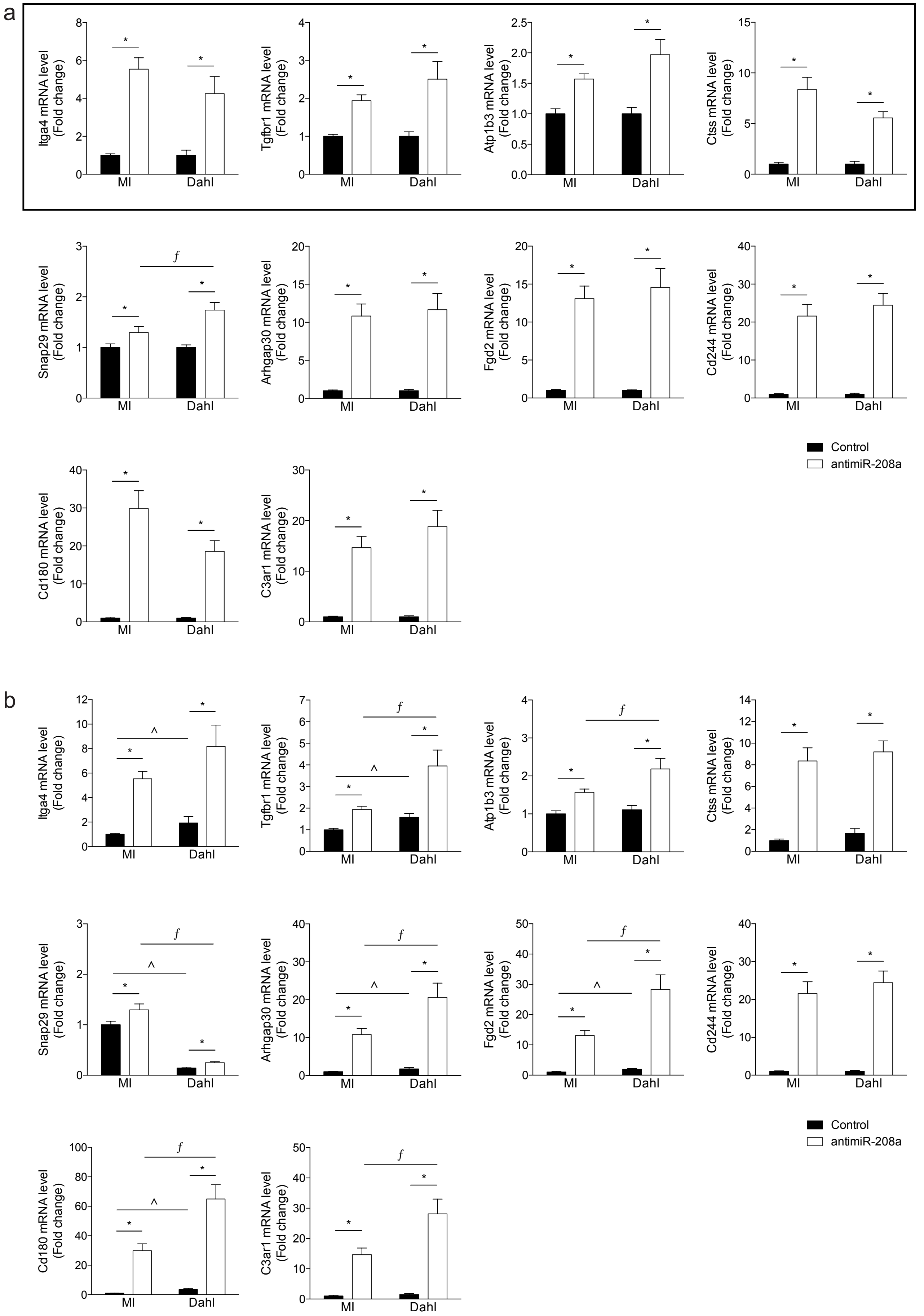

Figure S5. Real-time PCR analysis of miR-208a targets shown to be upregulated by gene array after antimiR-208a treatment both after MI surgery and in Dahl rats. (**a**) Data are expressed as mean fold change ± SEM for MI antimiR-208a (n=15-17) over MI control (n=17-19) and Dahl antimiR-208a (n=6-7) over Dahl control (n=5-6). (**b**) Data are expressed as mean fold change ± SEM for MI antimiR-208a (n=15-17), Dahl control (n=5-6) and Dahl antimiR-208a (n=6-7) over MI control (n=17-19). * indicates $p<0.05$ for antimiR-208a treatment versus control treatment; ^ indicates $p<0.05$ for control treatment between models; *ƒ* indicates $p<0.05$ for antimiR-208a treatment between models. Boxed graphs are shown in main figure 2f.

Figure S6

a

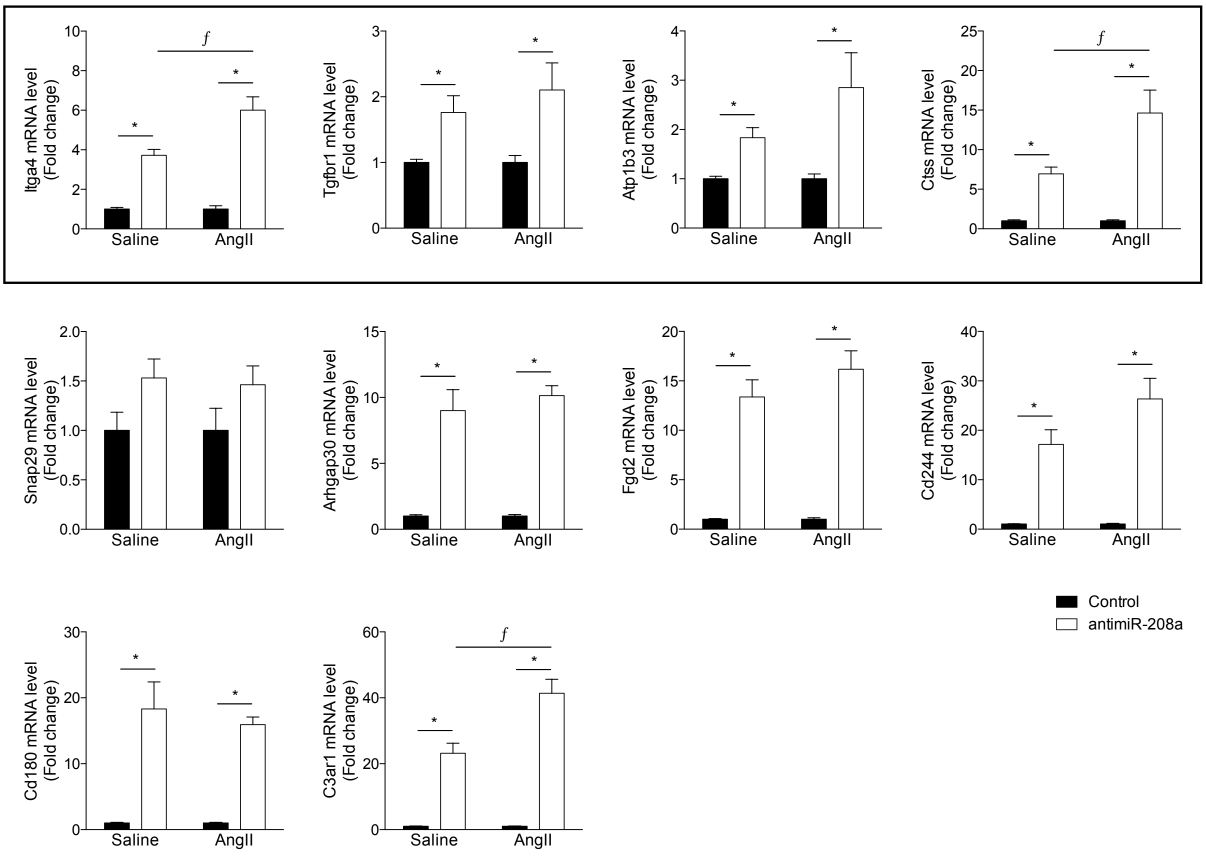

b

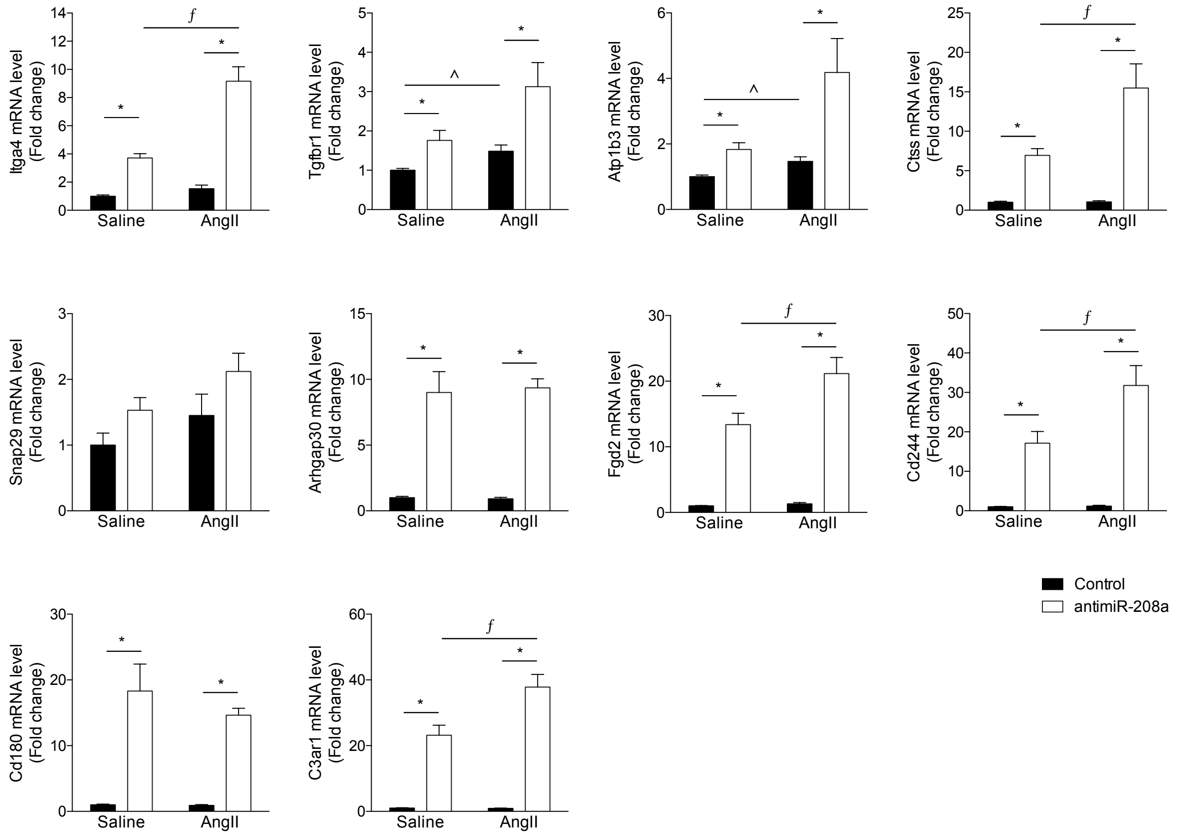

Figure S6. Real-time PCR analysis of miR-208a targets shown to be upregulated after antimiR-208a treatment both after MI surgery and in Dahl rats, determined in rats subjected to Angiotensin II (AngII) or vehicle (Saline) infusion prior to treatment with antimiR-208a or control. (**a**) Data are expressed as mean fold change ± SEM for saline antimiR-208a (n=5-6) over saline control (n=5-6) and AngII antimiR-208a (n=5-6) over AngII control (n=5-6). (**b**) Data are expressed as mean fold change ± SEM for saline antimiR-208a (n=5-6), AngII control (n=5-6) and AngII antimiR-208a (n=5-6) over saline control (n=5-6). * indicates $p<0.05$ for antimiR-208a treatment versus control treatment; ^ indicates $p<0.05$ for control treatment between saline and AngII; *ƒ* indicates $p<0.05$ for antimiR-208a treatment between saline and AngII. Boxed graphs were shown in main figure 2g.

Figure S7

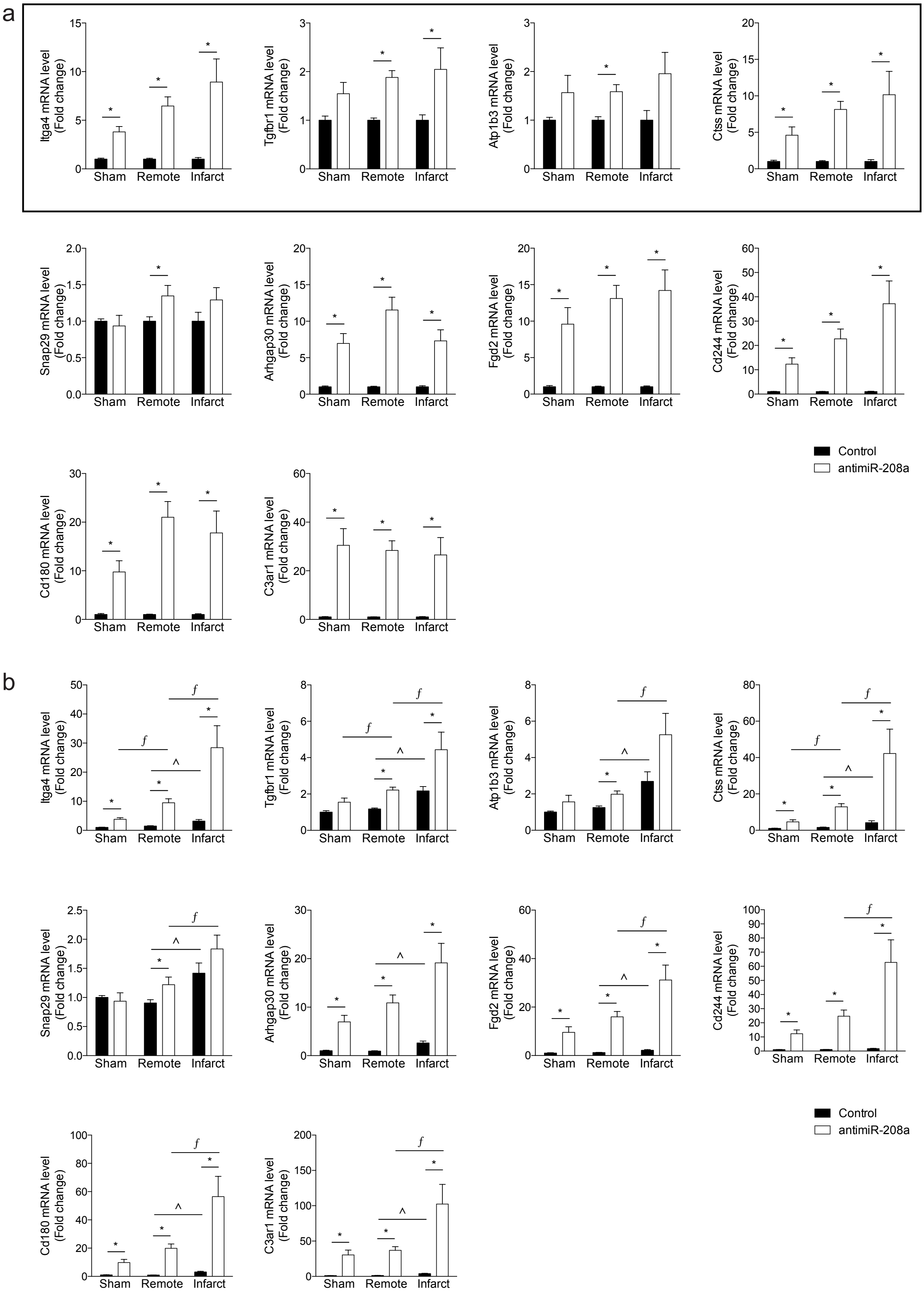

Figure S7. Real-time PCR analysis of miR-208a targets shown to be upregulated after antimiR-208a treatment both after MI surgery and in Dahl rats, determined in sham-operated hearts or different regions of the MI-operated hearts (Remote, Infarct) after control or antimiR-208a treatment. (**a**) Data are expressed as mean fold change ± SEM for Sham antimiR-208a (n=6-7) over Sham control (n=5-6), MI remote antimiR-208a (n=15-17) over MI remote control (n=17-19), and MI infarct antimiR-208a (n=15-17) over MI infarct control (n=17-19). (**b**) Data are expressed as mean fold change ± SEM for Sham antimiR-208a (n=6-7), MI remote control (n=17-19), MI remote antimiR-208a (n=15-17), MI infarct control (n=17-19) and MI infarct antimiR-208a (n=15-17) over Sham control (n=5-6). * indicates $p<0.05$ for antimiR-208a treatment versus control treatment; ^ indicates $p<0.05$ for control treatment between regions; *ƒ* indicates $p<0.05$ for antimiR-208a treatment between models. Boxed graphs were shown in main figure 3c.

Figure S8

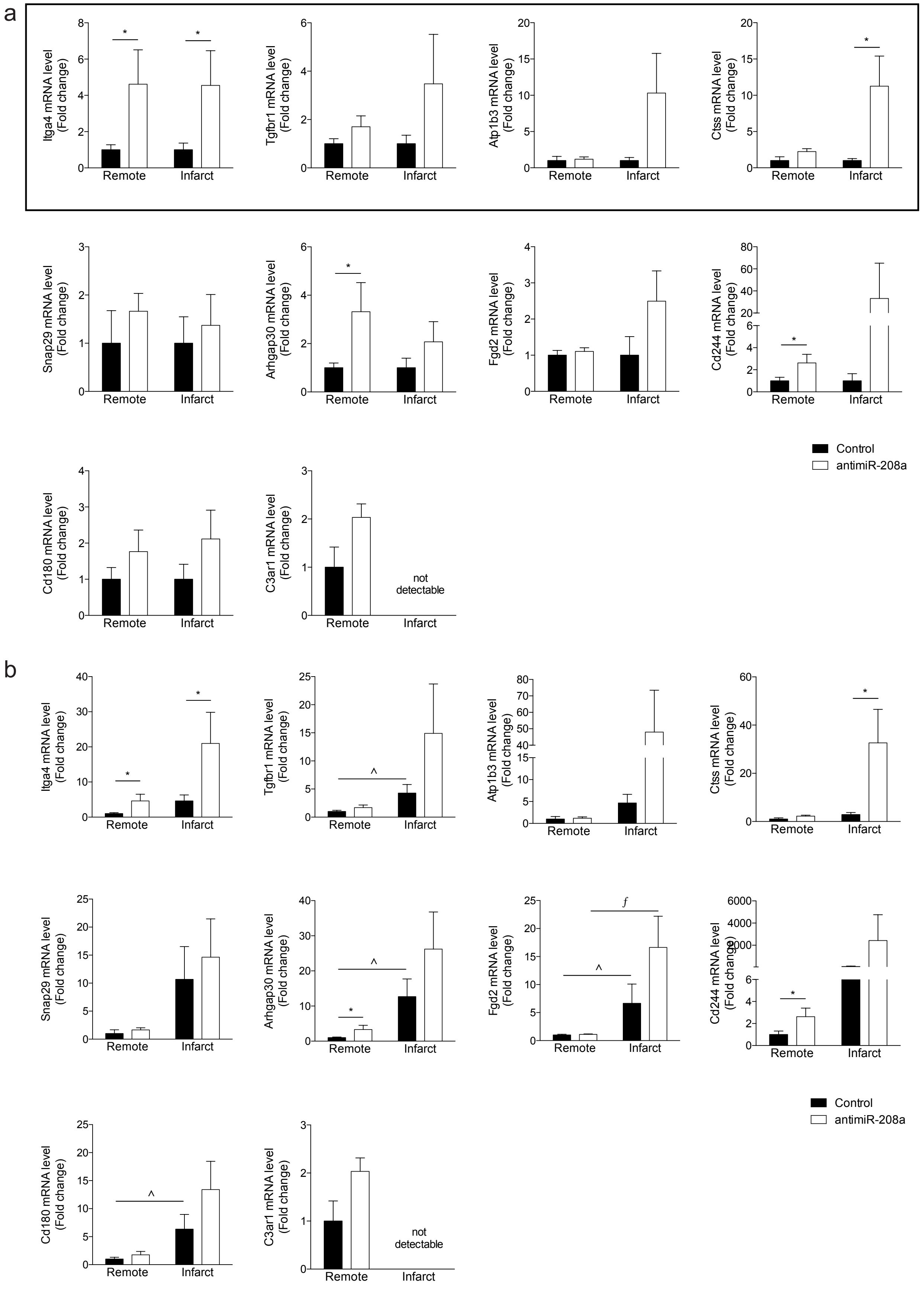

Figure S8. Real-time PCR analysis of miR-208a targets shown to be upregulated after antimiR-208a treatment both after MI surgery and in Dahl rats, determined in different regions (Remote, Infarct) of pig hearts subjected to ischemia-reperfusion injury with subsequent control or antimiR-208a treatment. (**a**) Data are expressed as mean fold change ± SEM for IR remote antimiR-208a (n=3-4) over IR remote control (n=6-7), and IR infarct antimiR-208a (n=3-4) over IR infarct control (n=5-6). (**b**) Data are expressed as mean fold change ± SEM for IR remote antimiR-208a (n=3-4), IR infarct control (n=5-6) and IR infarct antimiR-208a (n=3-4) over IR remote control (n=6-7). * indicates p<0.05 for antimiR-208a treatment versus control treatment; ^ indicates p<0.05 for control treatment between regions; *ƒ* indicates p<0.05 for antimiR-208a treatment between models. Boxed graphs were shown in main figure 3d.

Table S1. Fold change of seed-matched miR-208a target genes that are significantly upregulated in response to antimiR-208a after sham or MI surgery.

Table S1 data can be found as an Excel file in the online version of this manuscript.

Table S2. Fold change of seed-matched miR-208a target genes that are significantly upregulated in response to antimiR-208a after MI surgery, or in high salt diet fed Dahl rats.

Table S2 data can be found as an Excel file in the online version of this manuscript.

Table S3. List of real-time PCR primers

Rat primers

Gene	Species	Forward	Reverse
Map3k8	Rat (Rno)	CACGGAACACTCAGACTCCC	ATGTCCAGGACTTCCGACAC
Sash3	Rat (Rno)	CCCTGTCAGAGGAGATGGGA	TCACTGCCTGTTGATGTCTGG
Vcpip	Rat (Rno)	AGCCCACACTGTGAGACAAG	GACCACACGTCTTCTCCCATT
Prkcb	Rat (Rno)	ATGACCAAACACCCAGGCAA	GTGTCTCGCTTGTCTCTAGCTT
Evi2a	Rat (Rno)	GAGCGCTCTGTAAGATACCAGT	GGAACCCGAGGCAGTGATAC
Pip4k2a	Rat (Rno)	GACCATTACCAGCGAGGACG	CCGTCGACATTAAGCCGGTA
Dynlt1	Rat (Rno)	TCCACGGACGGGAGCTG	TAGAAACGCTGGAACCGGAG
Clecl4a3	Rat (Rno)	TCTCCAGTGCTCTGATCATTCTG	GGGCAACAGCTCCAGACTTT
GPR174	Rat (Rno)	ACCAAAAGGGCTGTGGTGTT	GGCCAGTCGTGGTTCAAGTA
Slc6a1	Rat (Rno)	CAGCCAGTTCTGTACCGTGG	GGCCAATCAGGTAGGACACG
Runx2	Rat (Rno)	CACAAGTGCGGTGCAAACTT	CCTTAAATATTACTGCATGGACTGT
Kif5a	Rat (Rno)	GCGCAGGAAACTGTACACGA	GTGATGGGCCTCACGATGAT
Cdh9	Rat (Rno)	AGGAAAGCTTCACACTGACCA	CTGTCCAGTTTCTTTGCGGC
Larp6	Rat (Rno)	CAAGAAGGTGAAACACCTCACAC	CCTTCCGGTGGTCCTCATTC
Cpox	Rat (Rno)	CTGAGGAGAGGGCGGTATGT	TCCCACCTTGCTGTTAGAGG
Klre1	Rat (Rno)	GGTCTGCCTTCTCCTGATGG	GGATGATGGAGTCCTTCCTGA
Plxnb2	Rat (Rno)	ACTATGACGAGATCATCAATGCTT	GCCACCCACCTGAATCCTG
Slc35b4	Rat (Rno)	GAAGCCACCAGCTATCCCAAT	GTTGGCAATCAGAGAGCCCGAT
Atp1b3	Rat (Rno)	CGAGTTAGTCCCAGATGGAGC	TGTCGATATCCAACGTGCCG
Snap29	Rat (Rno)	GCAGAACTAGACTCGGTCCC	CACGGACAGCTCATCTAGGTT
Tgfbr1	Rat (Rno)	AGAACTCCCAACTACAGAAAAGCA	ATGACAGTGCGGTTATGGCA
Fgd2	Rat (Rno)	GTGCCCAGTTCCAGGTGAG	CTGGCAGGCCTGTATCCAA
Itga4	Rat (Rno)	GCTGTTCACGGGTTTGTGAC	TTGGAGCCATGCTAATGCCA
Arhgap30	Rat (Rno)	TGTCCCAAACTACAGGACGC	AAGCCCGAGGCTTCTATGTC
Ctss	Rat (Rno)	GGGCAGCTGAAGCTGAAAAC	AAGCTTCGGTCATGAAGCCA
Cd244	Rat (Rno)	CGTCAAGAACGCACAAGTCAG	AAGACCTGTTGATGAACTGGAAAAG
Cd180	Rat (Rno)	CCTGGCACTCTACCAAACTCA	TCCAGTAAATCTGGCACCTGG
C3ar1	Rat (Rno)	TGCTCTTGACTGAGCAATGGA	TGCCTGGCAGTCCCAATAAA
Nppa	Rat (Rno)	AGGCCATATTGGAGCAAATC	CCTCATCTTCTACCGGCATC
Myh7	Rat (Rno)	GCTCACCTACCAGACAGAGGA	CAGGCATCCTTAGGGTTGGG
miR-208a	Rat (Rno)	ATAAGACGAGCAAAAAGCTTGT	GAATCGAGCACCAGTTACG
U6	Rat (Rno)	TGGCCCCTGCGCAAGGATG	GAATCGAGCACCAGTTACG

Pig primers

Gene	Species	Forward	Reverse
Atp1b3	Pig (Ssc)	TGGCCTCCTGGTTTGATCTT	TCCAACGCTGACACTGGTTT
Snap29	Pig (Ssc)	GACTCAGACTGCATCCCTGG	AGGGCAATGTCCTTCAGACG
Tgfbr1	Pig (Ssc)	GAGGCGACGGCATTACAGT	GGTCAATTTCAGCTATACACATGCT
Fgd2	Pig (Ssc)	TCAGGAGCCTGAGGAGAAGA	GCTCCTGGAAGAACACCTGG
Itga4	Pig (Ssc)	AACAGCTTCAGCTGGGTAGC	TGCCCACAAGTCACAATGGAT
Arhgap30	Pig (Ssc)	AGAGGCTTCAGGGTTCAACG	TTCCACCTCACCACCAGAGA
Ctss	Pig (Ssc)	AAGTAGCACGGCGTCTCATC	CACTGGTCATGTCTCCCAGG
Cd244	Pig (Ssc)	TCATGTTGAGAAGCCCCACC	CAGCTCACCCCCTTTGTACC
Cd180	Pig (Ssc)	TTGCTCGAGTCCTGTGATGG	CTGTTGGCTTCTTTCTCAGTGC
C3ar1	Pig (Ssc)	AGGTTTGGGACGTTTAGCCA	CCTGGCAACCCCAGTAAGAA
